# A Brief Resume of the Grosser Animal Nature and Its Application in Medicine

**Published:** 1901-04

**Authors:** G. N. Jack

**Affiliations:** Depew, N. Y.


### A BRIEF RESUME OF THE GROSSER ANIMAL NATURE AND ITS APPLICATION IN MEDICINE.[1]

By G. N. JACK, M. D., Depew, N. Y.

THE finer phenomena of animal nature or life and their results, are being and have been thoroughly studied through all phases from their origin to the completion of the circle of their existence, by the embryologist, the physiologist, the chemist, the anatomist and the pathologist.

These different scientists have nearly mastered the mechanism of animal life, while the grosser part of our animal nature, or the engineer that runs the engine, that has been so carefully studied as intelligence, including thoughts, desires, work, longings, ambitions and the termination of our existence, has been left to take care of itself, or to be governed by the ignorant and superstitious.

If, owing to the peculiar nature of our scientific studies, investigation and professional duties, we have been able to acquire positive knowledge as to the origin and conclusion of each individual animal life, such knowledge should be freely discussed at our medical societies, heated, hammered and forged into the scientific circle of life, thus beautifully completing it. Leaving this circle unforged not only mars the beauty of our science, but it is dangerous to it and greatly cripples the utility of our profession, for springing through this gap we behold such monsters as christian science, clairvoyancy, witchcraft, hoodooism and the masturbation quack.

There is much knowledge that the medical mind must be schooled to that would be harmful and dangerous in the hands of the average layman, but I have enough confidence in the members of my profession to believe that the thoughts here advocated will be kindly and confidentially received.

It is not the province of this paper to antagonise or belittle the church, that time-honored institution that has been directly instrumental in elevating humanity to the high plane it now occupies, but institutions that do not advance with civilisation must eventually be a detriment to intellectual progress. And today the harm done through the diseased imagination, arising from the teachings of some of the deluded and hysterical sects, is the production of a soil fertile to the growth of the christian science curse, is too graphically pictured before the medical mind for toleration.

1. Read at the annual meeting of the Medical Society of the County of Erie, January 8th and at the Central Medical Association, Lancaster, N. Y., February 4, 1901.

Christian science is so far removed from animal nature as to constitute a form of contagious insanity, and as it is our duty to protect communities against contagious diseases, it falls upon us to seriously consider this subject.

Being a member of the board of education of Depew, I can proudly say that I have been directly instrumental in weakening the medium through which this form of insanity develops, by instituting in our schools morning exercises consisting in a talk by the professor, discussing the natural and cultivated qualities most prominent in the production of noble lives, the reading of current topics and the singing of patriotic songs in lieu of devotional exercises, and it would be advisable to institute in some of our churches a similar mode of weekly instruction.

With all that might be said against Catholicism it, from a medical standpoint, has a redeeming quality in that it offers a sustaining power to suffering women that no other religious institution can ever hope to rival. For through the confessional weak women give vent to their natural animal feelings, and are ever encouraged by the priest, who is usually a well-read man, of good judgment, ever keeping abreast with the times. The result of this sort of vent to and constraining power over women, is most beautifully illustrated by the fact that any true Catholic woman would rather die than produce an abortion. It is also proverbially known that Catholic families have large numbers of children.

The physician, in that he continually meets undisguised nature in consultation, is in this respect placed in a similar attitude to that of the priest, and if the physician could exert over his non-catholic patients the same influence that the priest has over his people, he would be doing much toward stamping out the greatest crime of the age, childlessness.

Self-preservation and perpetuation is one of nature's greatest laws, and I have found it very useful in encouraging healthy, newly married people, to resolve to raise as large a number of children as they reasonably can. I never yet have failed to convince my patients that it was their most noble duty and that it should be the highest aim and greatest pride of their lives to raise, feed, clothe, educate and develop as many children as possible. It does not take one long, when armed with a few of nature's arguments, to convince his patients of these truths, for the frail, artificially constructed no-children ideas cannot, in the minds of those who have intelligence enough to conceive an idea, long resist the true steel of nature's laws when once brought face to face with them.

It is my custom to receive patients soliciting advice for the prevention of pregnancy kindly and sympathetically, thus gaining their confidence; I then proceed in the most simple manner possible, without in any way conflicting with their religious views, to prove to them, by explaining a few of nature's laws, that it is for their own selfish interest to raise as many children as possible, for it is through our progeny that nature secures for us perpetual life, with a most accurate preservation of all our notions, ideas, intellectual and physical development, peculiarities, and the like.

Then, as an illustration, to more forcibly impress upon their minds the point that I am trying to make, I usually tell them this story: When a boy down on the farm, a distant uncle once sent me two kernels of a rare and very choice variety of pop-corn. It is needless to say that these two kernels of corn were planted in the choicest earth, securely guarded and daily watched. When they reached the age of maturity and began to show evidences of earing, my childish curiosity could no longer forbear an investigation. The investigation proved disastrous to the tender budding ear of one stalk and in a few days it withered up and dropped to the ground. These two sister stalks, one pregnant and the other an abortionist, continued to grow side by side until old age caused them to naturally and resignedly part with that force known as life, and to gradually disintegrate into the chemical elements that composed them. As no one advocates a heaven or a hereafter for corn, it is evident then that the stalk of corn that aborted, in so doing forever lost its individuality and its only opportunity or hope of ever again participating in corn affairs; while the stalk that successfully bore the matured ear secured for itself as many chances for perpetual and improved life as there were healthy kernels on that ear.

After this corn story, I usually tell a similar story of some animal, as the horse, and gradually lead up to the life story of man and nature's mode of its perpetuation. Men are more willingly and easily convinced of the truth of these arguments than women, and for this reason it is always best, if possible, to have the husband present.

Many women, unless sustained by some convincing argument and the ardent desire of a respected husband, will take great chances, and repeatedly perform abortions upon themselves rather than endure the deprivations accompanying motherhood. One patient of mine, to my positive knowledge, performed an abortion upon herself three different times, and once upon her sister and a neighbor woman, and each of these five abortions was accompanied by retained putrifying secundi, that would soon have caused death were it not for the vigorous application of the curette.

Nature has wisely endowed the female of all animals with a strong, irresistible, sexual desire, which, if left to nature, will ever be periodically gratified until pregnancy supervenes. Education has taught women that the gratification of this passion usually produces pregnancy with its resultant pain, suffering, confinement, extra work in caring for the infant, and debarment from many of society's charms and advantages. It is a self-evident fact, then, that if a woman be endowed with the power to prevent pregnancy she will naturally gratify her passion, and naturally prevent pregnancy. For this reason no woman should ever be allowed to study or practise medicine, as it is placing her in an extremely dangerous position, alike from a personal, professional and national standpoint. The truth of these statements is proven beyond doubt, by the fact that few, if any, practising women physicians ever become mothers.

Owing to this peculiar nature of women our medical colleges are either intentionally or unintentionally doing an unchivalrous act in encouraging women to become physicians.

When the American government, like the French, become alarmed over the barrenness of her people, it will not take Yankee ingenuity long to place the responsibility, nor to contrive effective measures of reform, and when such a time comes, it would be far more honorable, and desirable, to be a galley slave than to unfortunately be affiliated with the designedly childless class.

It is also a physician's duty, when opportunity supervenes, to reach the innermost thoughts of boys and young unmarried men and thus ascertain whether or not they are, as is usually the case, while shunning an imaginary evil, gradually sinking into degeneration, infection, and corruption; if so, the physician should kindly show them their mistake and make them familiar with nature's laws.

Through the corrupt criminal and selfish teachings of the masturbation quacks who boldly use our mails and newspapers for the purpose of deceiving, many a well-meaning and sensitive youth of our country has been burdened with an imaginary sin to such an extent that he has been driven to drink, houses of immoral fame and suicide.

Many a high-spirited, well fed, healthy, full-blooded, robust youth has walked into my office, burdened by a mental condition bordering upon acute melancholia and with bowed head and tearful eye, proceeded to open up his life and finer sentiments to me for inspection and advice.

The story of these deceived youths has a marked similarity and usually runs as follows: He has frequently read in Dr. so and so's masturbation book, that if a young man masturbates or has wet dreams he will have lost manhood, weak memory, numerous other diseases and eventually go crazy. If he does not masturbate once a week or so, he will have wet dreams, he therefore is in a wretched condition, for he does not want to marry yet for several years; he knows the danger of having coition with immoral women, and they are disgusting to him; he has too much respect for woman hood to seduce a virtuous woman. He feels that there is no hope for himself, and that he is doomed to become a moral and mental wreck.

Such an unfortunate and self-respecting youth is deserving of our best and most considerate attention. Explain to them the character and object of the book, from which they have gotten their ideas; go minutely over the anatomy, physiology and function of the male generative organs, thus enabling them to understand that in the vigorous adult, they will naturally vent themselves once in ten or fifteen days.

Most young men of this nature will worry themselves nearly if not actually crazy, over masturbation practised once in two weeks or so. But if they have an opportunity to vent themselves on a female will have coition two or three times a day and feel that they are doing quite the proper thing. It does this class of men an immense amount of good and acts as a safety-valve to society and civilization, to let them know that masturbation, if practised with the same degree of prudence is no more harmful than coition.

Nature has so constructed the female of all animals that she does not desire, nor will not, if left to her own natural inclination permit coition, only at such periods as are most congenial to her conception.

They who have coition at these periods only, and if need be practise masturbation at other times to quell their passion, are much more true to nature's laws, and more honorable, than those who have coition time after time, utterly regardless of nature.

So long as physicians ignore these subjects and fail to reach the hearts of their patients, quacks will thrive.

(*Concluded in May Journal.*)

